# Animal experiment on osseointegration of porous titanium root analogue implants with composite CSn-TAK242 coating

**DOI:** 10.3389/fbioe.2025.1673758

**Published:** 2025-10-03

**Authors:** Hui Li, Dan Luo, Yudong Gao, Dashan Wang, Jianjun Yang, Zexian Xu

**Affiliations:** ^1^ The Affiliated Hospital of Qingdao University, Qingdao, China; ^2^ School of Stomatology, Qingdao University, Qingdao, China; ^3^ Dental Digital Medicine and 3D Printing Engineering Laboratory of Qingdao, Qingdao, China

**Keywords:** 3D printing, porous titanium, root analogue implants, osseointegration, chitosan

## Abstract

**Objective:**

Chitosan nanoparticles loaded with TLR4 inhibitors (TAK242) were coated on porous titanium root analogue implants and placeded into beagles to investigate the role of TLR4 inhibitors in inhibiting inflammatory reactions and promoting osseointegration *in vivo*.

**Methods:**

The control group consisted of porous titanium root analogue implants fabricated via digital medical technology and 3D printing, while the experimental group included porous titanium root analogue implants with CSn and CSn-TAK242 bioactive coatings. Three groups of implants were inserted into the jaws of dogs, with their stability coefficients immediately measured upon implantation. After 3 months, samples were collected, and the bone integration and gingival attachment of the three groups were assessed using X-rays, Micro-CT, and histological section staining.

**Results:**

All groups of porous titanium root analogue implants were correctly placed within the alveolar sockets. The stability coefficients of the implants immediately post-implantation in the control group, CSn group, and CSn-TAK242 group were (64.29 ± 4.01), (62.55 ± 1.98), and (64.59 ± 3.28), respectively, with no significant statistical difference (P>0.05). Three months post-surgery, imaging and histological examinations revealed bone integration with the surrounding bone tissue for all implant groups. BIC results showed: control group (68.11 ± 3.63)%, CSn group (71.07 ± 2.83)%, and Csn-TAK242 group (78.6 ± 4.52)%, with the BIC being highest in the CSn-TAK242 group, followed by the CSn group, and lowest in the control group (P<0.05). More importantly, compared with the control group, the BV/TV of the CSn-TAK242 group was significantly higher. In addition, the Tb.Th of the CSn-TAK242 group was significantly higher than that of the control group and CSn group (P<0.05). The smooth structures at the upper ends of the implants had tight gingival tissue attachment.

**Conclusion:**

Porous titanium root analogue implants consistent with the target root morphology were successfully fabricated using digital medical technology and 3D printing. The composite CSn-TAK242 coating further enhanced the osseointegration effects of these implants.

## 1 Introduction

Implant-supported dental prostheses are preferred for their aesthetic appeal, comfort, and high success rates, making them the optimal restoration method for patients with missing teeth (Wu et al., 2021). However, the standard cylindrical or conical shapes commonly used for implants in oral clinics differ significantly from the natural form of tooth roots, especially in the molar region, leading to compromised initial stability (Croitoru and Inţă, 2019; Zhao et al., 2025). Additionally, traditional titanium implants often face challenges related to their high elastic modulus, which can cause stress shielding and low bioactivity, thereby increasing the risk of surgical failure (Dallago et al., 2018; Li et al., 2020).

To address these limitations, researchers have designed and manufactured personalized titanium root analogue implants (RAI) that mimic the natural root morphology. RAI adapts better to the shape of the extraction socket, minimizes damage to surrounding bone and soft tissues, and simplifies the surgical process as it does not require the implantation of bone substitutes and allows for immediate placement (Dantas et al., 2021; Aldesoki et al., 2024). With the rapid advancement of 3D printing and computer-aided design/manufacturing (CAD/CAM) technologies, some scholars have created porous RAI with a bone-like trabecular structure (Aldesoki et al., 2023). Studies indicate that the porous design of these implants facilitates metabolic waste exchange. Additionally, implants with a porosity of 60%–75% have an elastic modulus similar to bone, which enhances osteoblast adhesion and promotes osseointegration (Schubert et al., 2018; Böse et al., 2020).

The integration of the implant with surrounding bone tissues is crucial for the success of oral implantation (Bohara and Suthakorn, 2022). In the initial phase of osseointegration, mild inflammatory responses induced by tissue damage and cellular debris are essential for activating bone resorption and new bone formation. However, excessive inflammation and microbial infection can impair normal bone healing. DAMPs, such as cell debris and necrotic tissue products, can be recognized by Toll-like receptor 4 (TLR4), leading to the translocation of NF-kB and the release of cytokines, further exacerbating the inflammatory response and inhibiting tissue repair (Al-Rikabi et al., 2020; Xu et al., 2020). An abundance of inflammatory cytokines stimulates the proliferation and differentiation of osteoclasts, accelerating bone resorption, which may eventually lead to implant failure (Gao et al., 2015). Thus, reducing inflammation early in bone healing is beneficial for implant osseointegration.

According to recent studies, TLR4 is essential in mediating inflammatory responses and regulating bone metabolism (Jin et al., 2023). Activation of TLR4 stimulates the Medullary Differentiation Factor 88 (MyD88) signaling pathway, regulating osteoclast differentiation. Inhibiting TLR4 expression can promote cell proliferation and inhibit apoptosis, thereby accelerating the bone healing process (Kong et al., 2024; Tominari et al., 2024; Park et al., 2025). A study on cranial defect repair in mice genetically modified to lack TLR2 and TLR4 demonstrated superior bone healing performance compared to normal mice, further confirming the critical role of TLR4 in bone regeneration (Chukkapalli et al., 2018).

Our previous research found that chitosan nanoparticles (CSn) can serve as an effective drug delivery system (Zhou et al., 2021). CSn loaded with the TLR4 inhibitor TAK242 (CSn-anti-TLR4) has been shown to suppress inflammatory responses and promote cell proliferation and osteogenesis, offering a new therapeutic strategy to enhance alveolar bone healing and, consequently, implant osseointegration (Bhattacharyya et al., 2018). However, studies exploring the role of TLR4 inhibitors in enhancing implant osseointegration in animal models are currently lacking.

Therefore, this study employs digital medicine and 3D printing technology to fabricate personalized titanium RAIs that match the target tooth roots of Beagle dogs. The surface of these implants is coated with CSn and CSn loaded with the TLR4 inhibitor TAK242 (CSn-TAK242), to observe the osseointegration of RAIs in Beagle dogs. This research aims to provide evidence for the clinical application of composite-coated porous titanium RAIs and offer new insights into improving clinical implant success rates and lifespan.

## 2 Materials and methods

### 2.1 Experimental animals

The experimental animals were provided by Qingdao bolong Beagle Dog Breeding Co.,Ltd. Six healthy adult male Beagle dogs aged between 1 and 1.5 years, with an average weight of (15.2 ± 0.6) kg, were selected. All animal experiments were conducted in accordance with the guidelines for animal laboratory use and the operating regulations of the Animal Ethics Committee of Qingdao University Affiliated Hospital (NO. QYFYKYLL 958311920).

### 2.2 Main instruments and materials

Materials and equipment used included Ti_6_Al_4_V powder with a purity of 99.7% and particle size of 20–50 μm (Beijing Zhonghang Maite Powder Metallurgy Technology Co., Ltd., China), chitosan powder (Shandong Hengtai Jinhu Biological Products Co., Ltd., China), TAK-242 (Apoptosis and Epigenetics Company, United States), I-CAT CBCT tomography system (CAVA, United States), Mimics 17.0 (Materialise, Belgium), Geomagic Studio modeling software (Materialise, Belgium), MLAB R metal 3D laser printer (Concept laser, Germany), scanning electron microscope (SEM, JEOL, Japan), X ray diffraction (XRD, X'Pert Pro MPD, PANalytical), Fourier transform infrared spectrometer (FTIR, Nicolet 380 IR, Thermo Scientic), Micro-CT scanner (SCANCO Medical AG, Switzerland), among others.

### 2.3 Preparation and grouping of the composite coating on the 3D printed porous titanium RAIs

#### 2.3.1 Data acquisition and modeling

**FIGURE 1:**
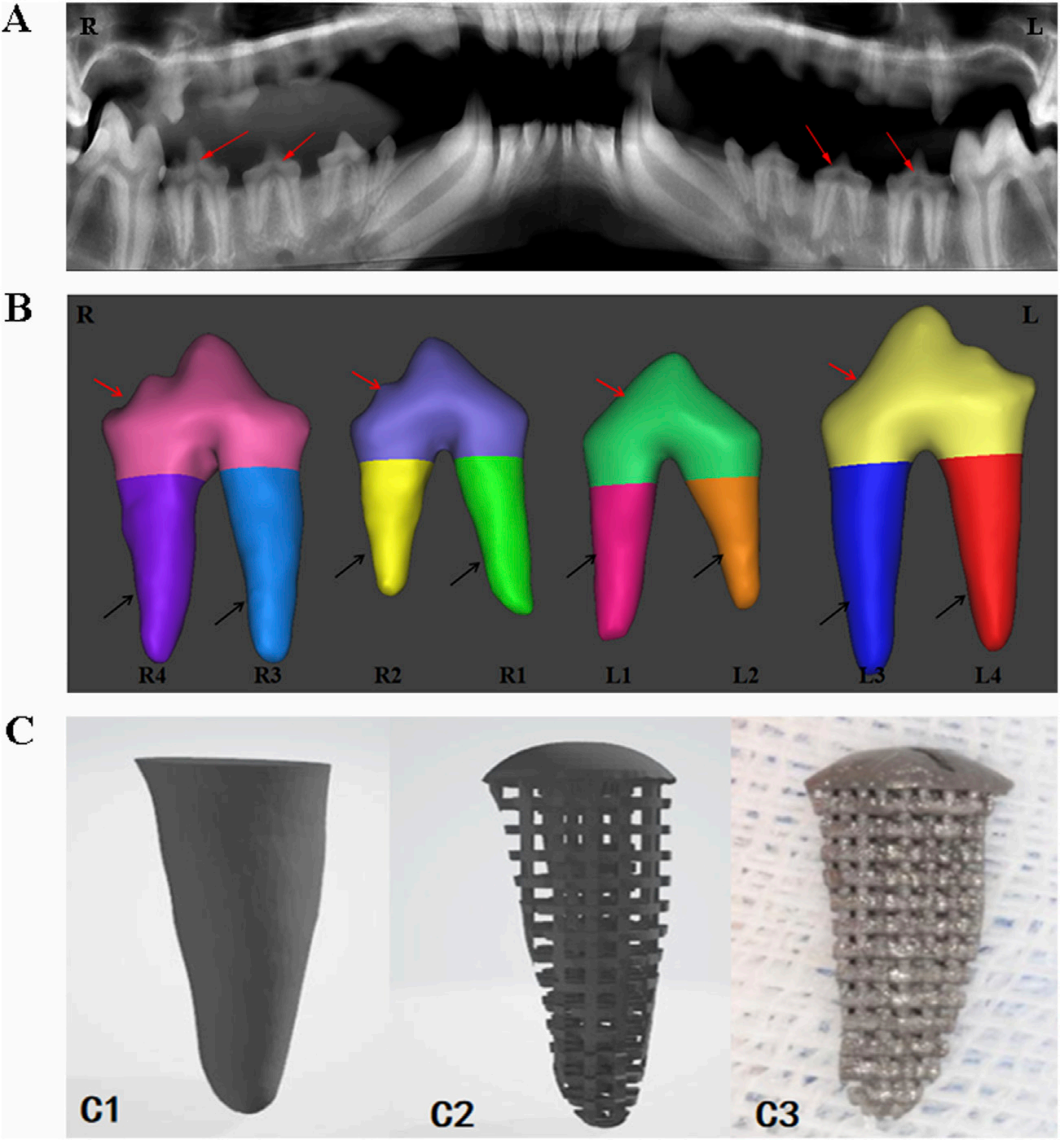
Digital model acquisition, design, and printing of RAIs. **(A)** CBCT data acquisition. **(B)** 3D model of target tooth, the red arrow indicates the crown part, and the black arrow indicates the root part. **(C1)** Extract the target tooth root image. **(C2)** Constructing a three-dimensional digital model of porous structures. **(C3)** 3D printing of porous titanium RAIs that match the shape of the target tooth root.

The six Beagle dogs were sequentially numbered from D1 to D6. After anesthesia, they were fixed in the CBCT scanning chamber for sequential scanning (Figure 1A), and the original image data in DICOM format was exported. The data was imported into Mimics 17.0 software, where the bilateral third and fourth premolar roots of the six Beagle dogs (a total of 24 teeth, 48 roots) were selected as target roots and numbered (Figure 1B). Appropriate thresholds were set to extract three-dimensional images of the target roots. The upper part of the tooth crown was removed at the alveolar ridge crest, and the extracted three-dimensional images were imported into Geomagic Studio software for designing the porous structure of the RAIs and the rounded structure at the upper end of the roots (Figure 1C_1_C_2_).

#### 2.3.2 Preparation of porous titanium RAIs

The digital model data in STL format was imported into the MLAB R metal 3D laser printer, which used Ti_6_Al_4_V powder with a particle size of 20–50 μm to perform melting and sintering in an argon environment. The laser spot diameter was 70 μm, the powder layer thickness was 50 μm, and the production speed was 80 cm^3^/h. Forty-eight porous titanium RAIs matching the morphology of the target roots were manufactured based on the modeling design (Figure 1C_3_).

#### 2.3.3 Preparation and grouping of the composite coating on the surface of the porous titanium porous titanium RAIs

This experiment employed the coating preparation methods for CSn and CSn-TAK242 previously established in earlier *in vitro* studies. Specifically,100 mg of CS powder was dissolved in 0.1 mol/L acetic acid solution, which was then brought to a final volume of 100 mL. The mixture was stirred continuously for 1 h using a magnetic stirrer to obtain a 1 mg/mL chitosan solution. An appropriate amount of this chitosan–acetic acid solution was taken, and a 5% (w/v) sodium tripolyphosphate solution was added dropwise under continuous magnetic stirring until the solution turned turbid. Stirring was continued for another 30 min, followed by standing for 15 min. The supernatant was collected and subjected to sonication for 5 min to obtain a well-dispersed CSn suspension. For the preparation of CSn-TAK242, an appropriate volume of the 1 mg/mL chitosan–acetic acid solution was mixed with TAK242 dilution to achieve a final drug concentration of 2 μM. To this chitosan–TAK242–acetic acid mixture, a 5% sodium tripolyphosphate solution was added dropwise under magnetic stirring until turbidity appeared. The mixture was stirred for another 30 min, allowed to stand for 15 min, and then the supernatant was collected and sonicated for 5 min to yield a uniformly dispersed CSn-TAK242 solution. By the ion crosslinking method, CSn and CSn-TAK242 solutions were prepared, and the porous titanium RAIs (n = 16) were immersed in CSn and CSn-TAK242 solutions for 10 min, air-dried naturally, and this step was repeated three times to obtain a uniform coating density. These were respectively labeled as the CSn group and the CSn-TAK242 group. Implants without surface coating treatment were designated as the Control group.

### 2.4 Characterization

The surface morphology of the three groups of implants was observed using SEM. The elemental composition of the implants was detected using XPS. Phase analysis of the samples was conducted using XRD at a scanning speed of 10°/min and a scanning range of 10°–80°. The structural analysis was performed using FTIR within the testing range of 4,000–400 cm^-1^.

### 2.5 Animal experiment

**FIGURE 2:**
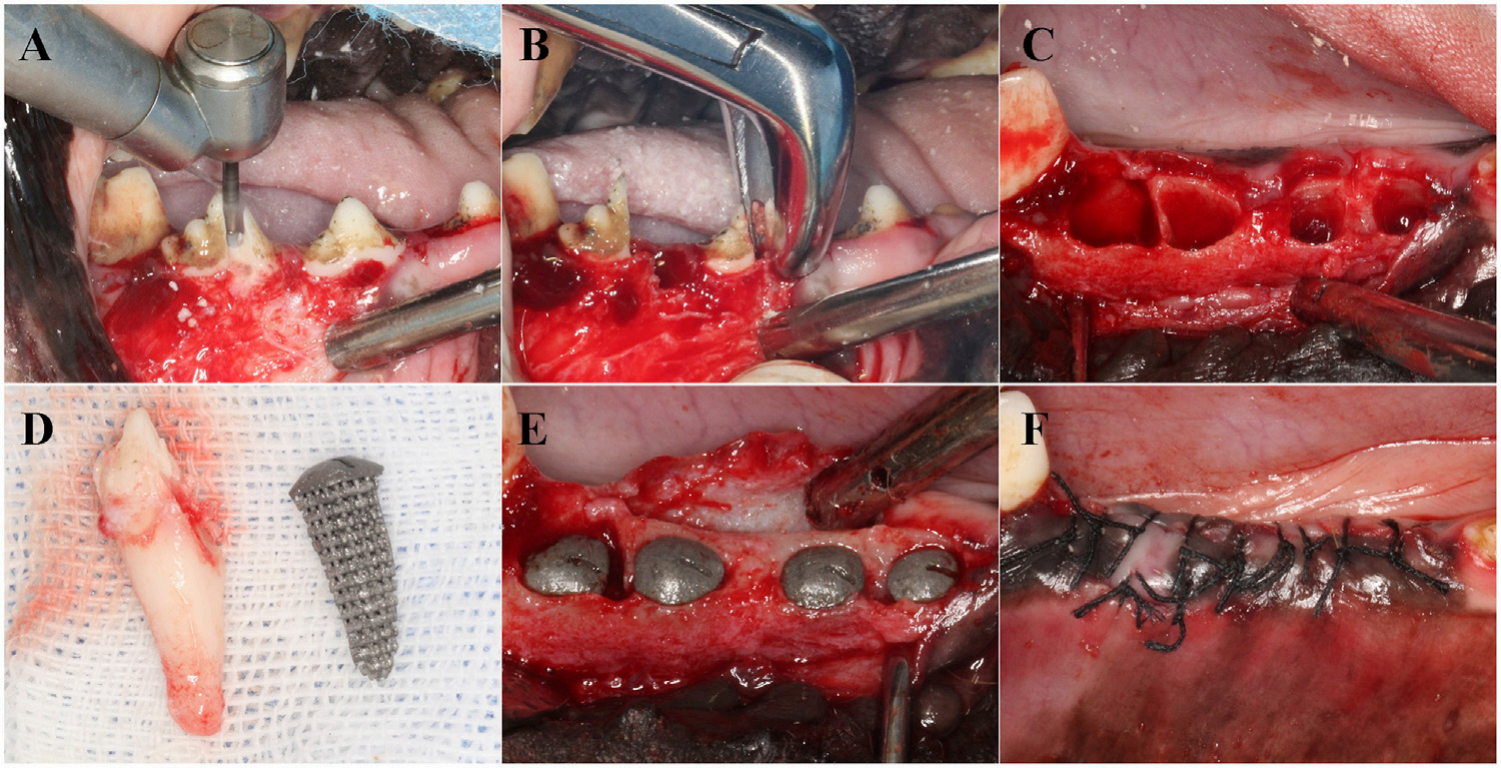
Implantation process of RAIs. **(A)** Separate dental crowns. **(B)** Remove the root of the teeth. **(C)** Scratching and cleaning the alveolar cavity. **(D)** RAIs consistent with the target teeth. **(E)** Place the RAIs in the corresponding position. **(F)** Suture.

The entire surgical procedure was performed under general anesthesia induced using intravenous propofol (2–5 mg/kg/i.v.) and maintained with isoflurane (1%–3%). The dogs were first premedicated with medetomidine (30 μg/kg/i.m.). Local anesthesia at the surgical sites was induced by injecting 2% lidocaine hydrochloride with 1:100,000 epinephrine. The Beagle dogs were secured on the operating table, and after disinfection, the third and fourth premolars were minimally extracted. The sterilized RAIs from the three groups were implanted into the corresponding alveolar sockets according to their numbers. A Summers bone chisel was used to position the implants along the longitudinal axis of the tooth root, and a bone hammer was lightly tapped to position them. The immediate implant stability quotient (ISQ) was measured using the ID 5 implant stability measurement device. The surgical site was sutured tightly to achieve submerged healing (Figure 2). The retention and growth of the implants within the Beagle dogs were observed periodically.

### 2.6 Radiographic observation

Three months post-surgery, the dogs were sedated with medetomidin and anesthetized with isoflurane and propofol. Pentobarbital overdose (40–60 mg/kg/i.v., Dolethal, Vetoquinol) was used to euthanize the dogs. Mandibular bone tissue blocks containing a single RAI and surrounding bone tissue of approximately 5 mm were excised. The tissue blocks were immersed in 4% polyformaldehyde solution and fixed for 24 h before taking X-ray images to observe the status of the RAIs within the tissue blocks. The bone density of the internal and external 1 mm thick regions of the simulated RAIs were measured, and the average value was calculated. The bone density measurement results were expressed in Hounsfield units (HU). Concurrently, Micro-CT scanning was performed, and the new bone formation volume/total volume ratio (BV/TV), trabecular thickness (Tb.Th) and the bone-to-implant contact (BIC) for the three groups of RAIs were analyzed.

### 2.7 RT-qPCR detection

Three months post-surgery, under general anesthesia, the Beagle dogs were euthanized, and bone tissue around the implants from each group was excised. RNA from the bone tissue near the implants was extracted, and the expression of cytokines in the alveolar bone around the implants was measured using RT-qPCR (n = 3). The extracted RNA was reverse-transcribed into cDNA using the HiScript II Q RT SuperMix for RT-qPCR (gDNA wiper) kit. The expression levels of MyD88 mRNA, TRIF mRNA, TLR2 mRNA, and TLR4 mRNA in the bone were detected using an RT-qPCR instrument. The primer sequences are shown in Table 1, with the β-actin gene serving as an internal reference.

**TABLE 1:** RT-qPCR primer sequence.

Primer	Sequence
MyD88	CCAGGGGGTCTGTATGCTTG CCCCAGACCCTGAGAAAAGG
TRIF	TGCACTGAAATGTTGGCAGC AACTGGGAAGCGGTGTCTTC
TLR2	AGCCTTGACCTCTCCAACAA TGAGGTTCACACAATCCCGA
TLR4	GTTTGAAGCAGGCCAGTGAT GGCTGACCAAGCCATCAAAA
β-actin	TGTGTTATGTGGCCCTGGAC TTCCATGCCCAGGAAGGAAG

MyD88, myeloid differentiation primary response gene 88; TRIF, TIR-domain-containing adaptor inducing interferon-β; TLR2, Toll-like receptor 2; TLR4:Toll-like receptor 2.

### 2.8 Histomorphological analysis

The fixed tissue blocks were rinsed with saline and dehydrated using a gradient of alcohol concentrations at 50%, 75%, 85%, 95%, and 100%. The blocks were then infiltrated and embedded with light-curing resin. Slices were cut along the buccolingual longitudinal axis of the tissue blocks using an EXAKT hard tissue cutting machine, with a slice thickness of 50 μm. The slices were then ground using the EXAKT grinding system and subsequently stained with Ladewig and encapsulated to produce histological sections, which were observed under an optical microscope to examine the bone tissue growth into the pores of the porous titanium RAIs and the attachment of the smooth rounded structures at the upper end of the implants to the gingival tissues.

### 2.9 Statistical analysis

Data analysis was performed using SPSS 22.0 statistical software. The normality of the data was confirmed by the Shapiro-Wilk test, and homogeneity of variances was verified using Levene’s test. Continuous data are presented as X ± S deviation. An independent samples t-test was employed to compare the data between the three groups, with P<0.05 considered statistically significant.

## 3 Results

### 3.1 Characterization

**FIGURE 3:**
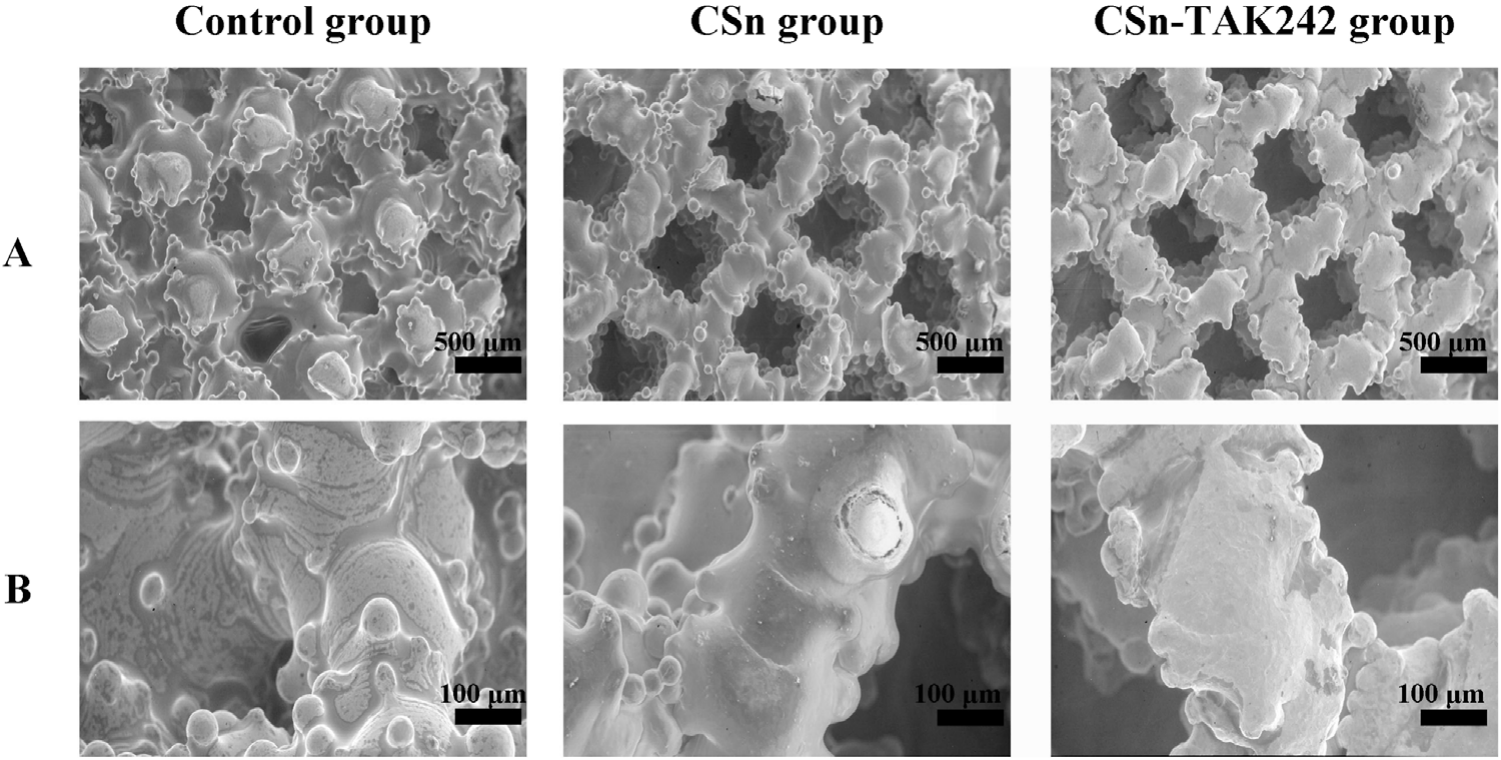
SEM images of three groups of RAIs. **(A)** Low magnification. **(B)** High magnification.

The surfaces of the three groups of implants were observed using scanning electron microscopy after fixation. Under low magnification (Figure 3A), the implants appeared as layers of mesh structures accumulated with some titanium particles fused and protruding in a semi-spherical shape, creating a rough surface. The different meshes intertwined and connected with each other. The surface porosity of the three groups was similar, each featuring pores ranging from 300 to 500 μm in size, with good interconnectivity. Under high magnification (Figure 3B), the surface of the control group was smooth and rounded, while the experimental groups exhibited a uniformly distributed and dense coating.

**FIGURE 4:**
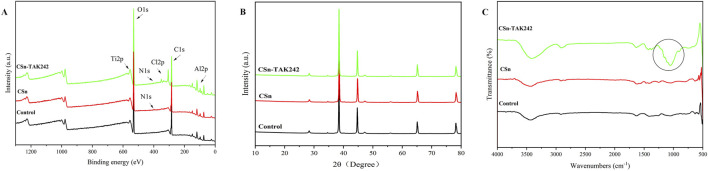
Characterization of three groups of RAIs. **(A)** XPS, **(B)** XRD and **(C)** FTIR.

XPS results (Figure 4A) showed that the Control group contained only titanium alloy elements Ti and Al, the CSn group included the addition of N, and the CSn-TAK242 group also showed traces of Cl, an element from the TAK242 drug. XRD patterns (Figure 4B) demonstrated that, compared with the Control group, no additional peaks emerged in the XRD profiles of the CSn group and the CSn-TAK242 group. The FTIR results (Figure 4C) revealed a distinct peak at 1,054 cm^-1^ in the CSn-TAK242 group, which corresponds to the characteristic peak of the TAK242 drug, while the other peaks exhibited similarity among the three groups. Through the analysis of the above results, it can be confirmed that CSn-TAK242 has successfully polymerized on the surface of the RAIs.

### 3.2 Postoperative observations

All 48 porous titanium RAIs prepared using 3D printing technology were successfully placed in the alveolar sockets. As shown in Table 2, the ISQ results were as follows: Control group (64.29 ± 4.01), CSn group (62.55 ± 1.98), and CSn-TAK242 group (64.59 ± 3.28), with no significant statistical difference in implant stability quotient among the three groups (P>0.05). The gingival flaps were able to completely cover the top of the implants and were tightly sutured closed. Ten days after surgery, some sites showed suture loss, wound dehiscence, and gingival swelling with the structure of the top of the porous titanium RAIs exposed but stable. All other wounds healed primarily with no signs of gingival swelling or infection. Three months post-implantation, CBCT imaging showed that 7 implants were lost, with 14 remaining in the CSn-TAK242 group, 14 in the CSn group, and 13 in the Control group. The remaining 41 porous titanium RAIs were analyzed.

**TABLE 2:** ISQ, Survival rate, HU values, BV/TV, Tb.Th, BIC of three groups of RAIs.

Project	Control group	CSn group	CSn-TAK242 group
ISQ values	64.29 ± 4.01	62.55 ± 1.98	64.59 ± 3.28
Survival rate (%)	81.25%	87.5%^a^	87.5%^a^
HU values	276 ± 11.43	293 ± 7.92	423 ± 15.74^a^ ^,^ ^b^
BV/TV (%)	53.62 ± 2.74	60.97 ± 2.68^a^	68.58 ± 3.14^a^ ^,^ ^b^
Tb.Th (mm)	0.3435 ± 0.0018	0.3709 ± 0.0026^a^	0.4406 ± 0.0032^a^ ^,^ ^b^
BIC (%)	68.11 ± 3.63	71.07 ± 2.83	78.69 ± 4.52^a^ ^,^ ^b^

Statistical differences are represented as.

^a^ P < 0.05 (vs. Control group).

^b^ P < 0.05 (vs. CSn, group).

### 3.3 Radiographic observation

**FIGURE 5:**
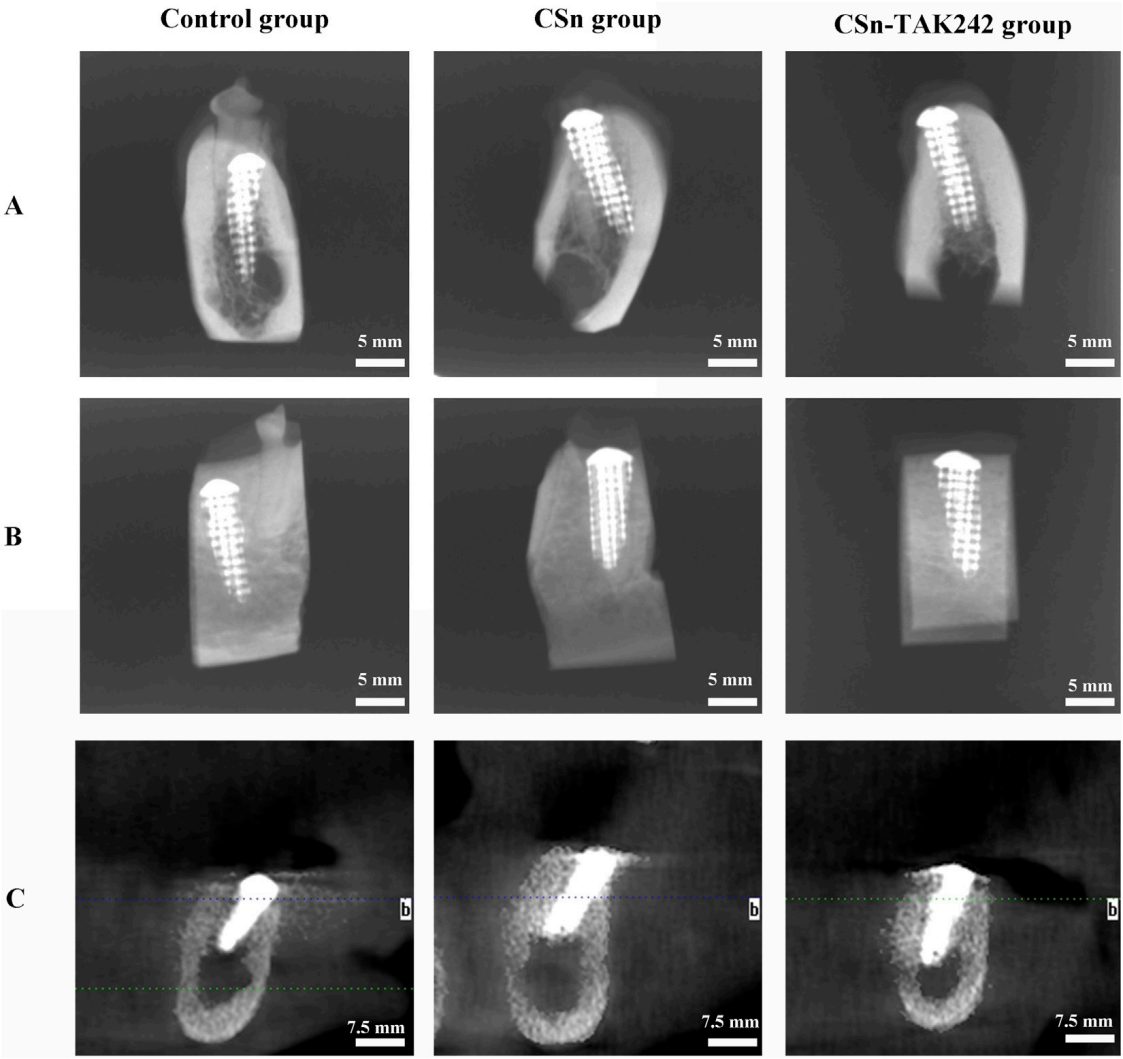
Radiographic observation of three groups of RAIs. **(A)** Buccal and lingual diameters of X-rays. **(B)** Mesial and distal diameters of X-rays. **(C)** CBCT.

**FIGURE 6:**
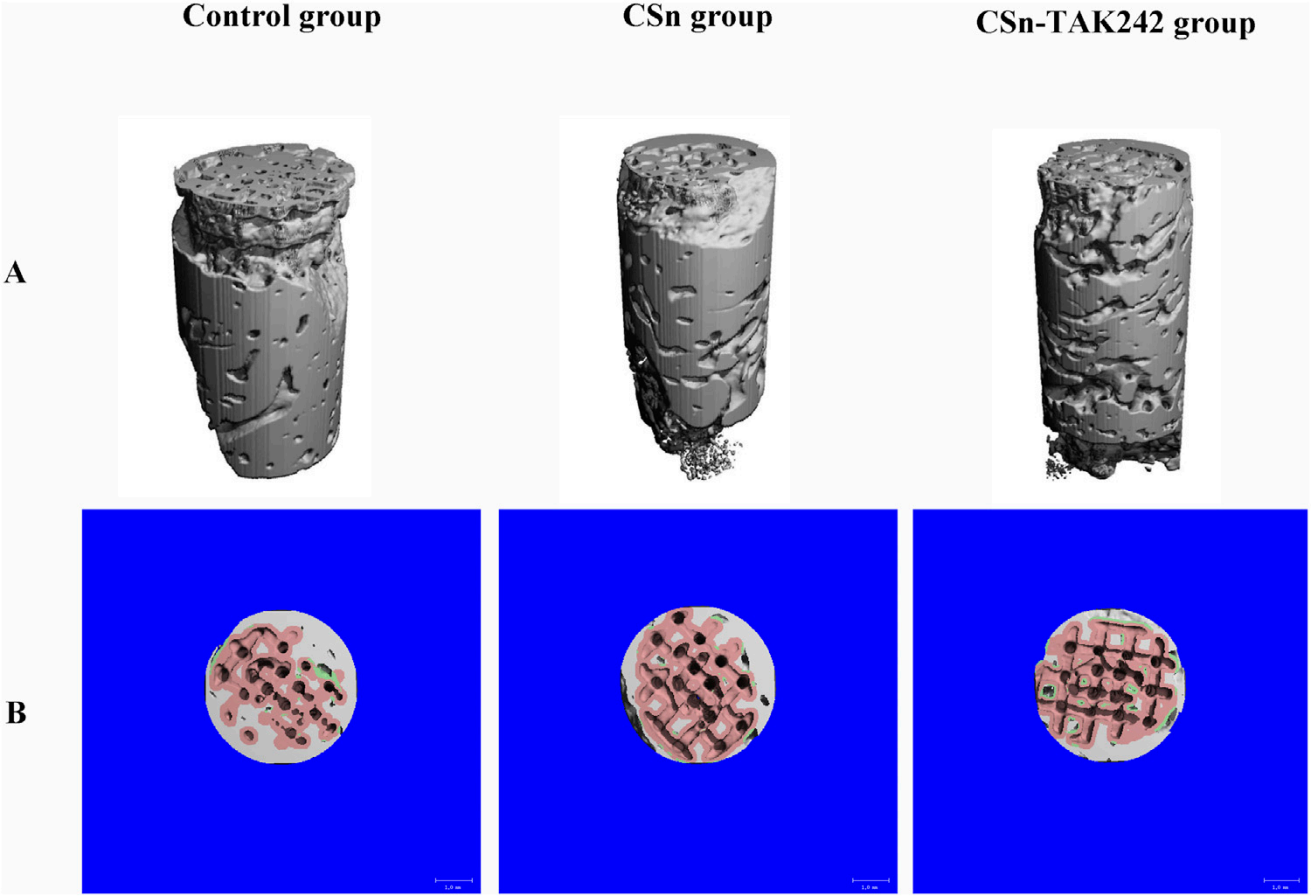
Micro CT image of three groups of RAIs at 3 months post-surgery. **(A)** 3D image. **(B)** Cross section plane image.

As shown in Figure 5, X-rays indicated that all three groups of porous titanium RAIs were within the alveolar bone and tightly integrated with it, with no transmission shadow between the implant and bone, and no significant bone resorption. As shown in Table 2, HU values for the CSn-TAK242 and CSn groups were higher than those for the Control group, with the CSn-TAK242 group showing higher values than the CSn group (P<0.05). Micro-CT scans demonstrated that the pores of the porous titanium RAIs were filled with substantial bone tissue and closely integrated with the implants (Figure 6). BIC results showed: Control group (68.11 ± 3.63), CSn group (71.07 ± 2.83), and Csn-TAK242 group (78.69 ± 4.52), with the BIC being highest in the CSn-TAK242 group, followed by the CSn group, and lowest in the Control group (P<0.05). More importantly, compared with the Control group, the BV/TV of the CSn-TAK242 group was significantly higher. In addition, the Tb.Th of the CSn-TAK242 group was significantly higher than that of the Control group and CSn group, indicating that the CSn-TAK242 group had more thickness and quantity of new bone trabeculae, which demonstrated that the CSn-TAK242 group has a better effect on implant osseointegration (Table 2).

### 3.4 Cytokine mRNA expression in bone tissue

**FIGURE 7:**
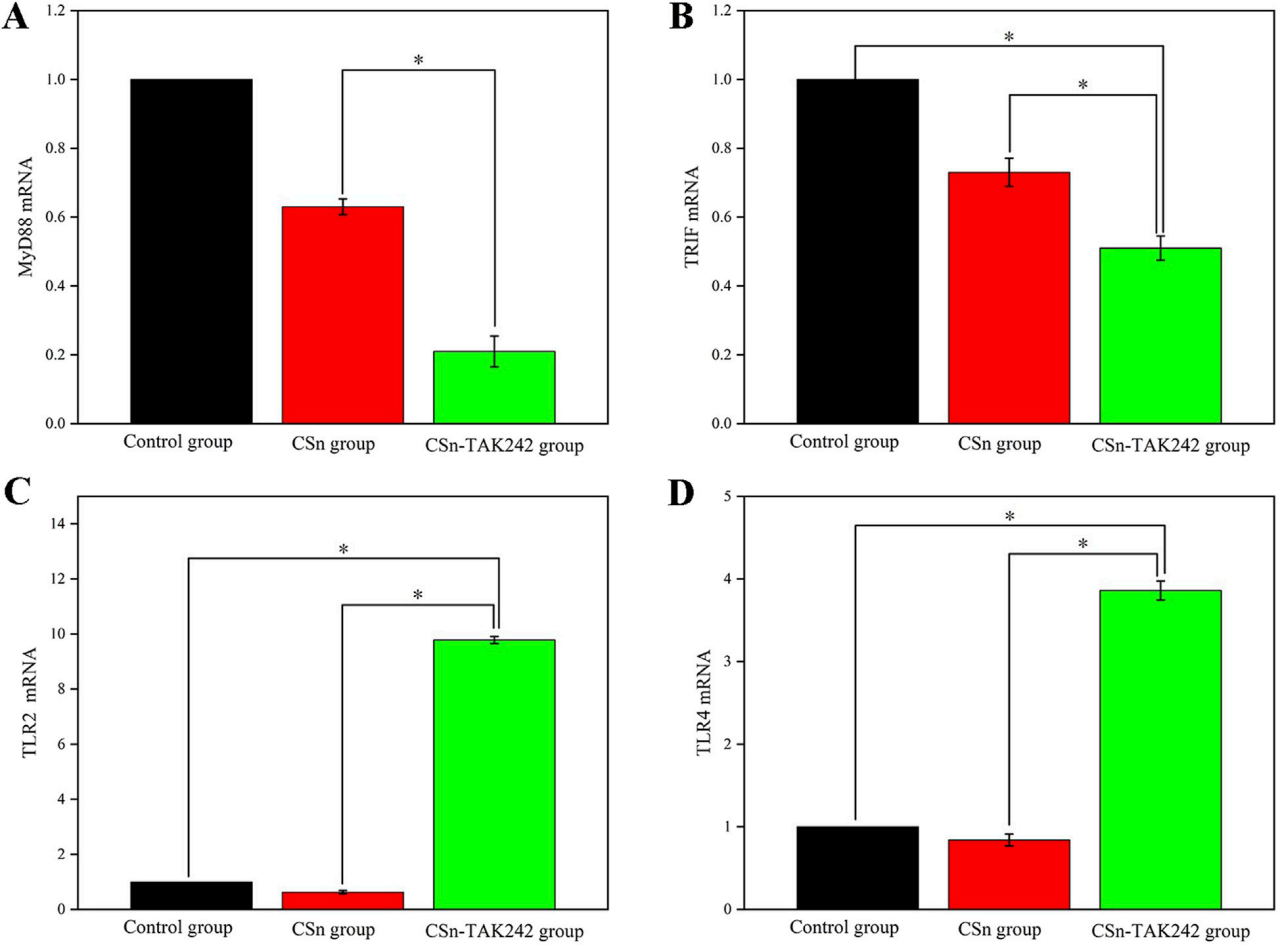
Cytokine mRNA expression in bone tissue (*p < 0.05). **(A)** MyD88 mRNA. **(B)** TRIF mRNA. **(C)** TLR2 mRNA. **(D)** TLR4 mRNA.

mRNA was extracted from bone tissue, and the expression of cytokines in peri implant alveolar bone was detected by RT-qPCR. Compared to the CSn and Control groups (Figures 7A,B), the CSn-TAK242 group showed significantly reduced expression of MyD88 mRNA and TRIF mRNA (P<0.05). The CSn-TAK242 group exhibited significantly increased expression of TLR2 mRNA and TLR4 mRNA compared to the Control and CSn groups, with no significant differences between the Control and CSn groups (Figures 7C,D).

### 3.5 Histological slice observations

**FIGURE 8:**
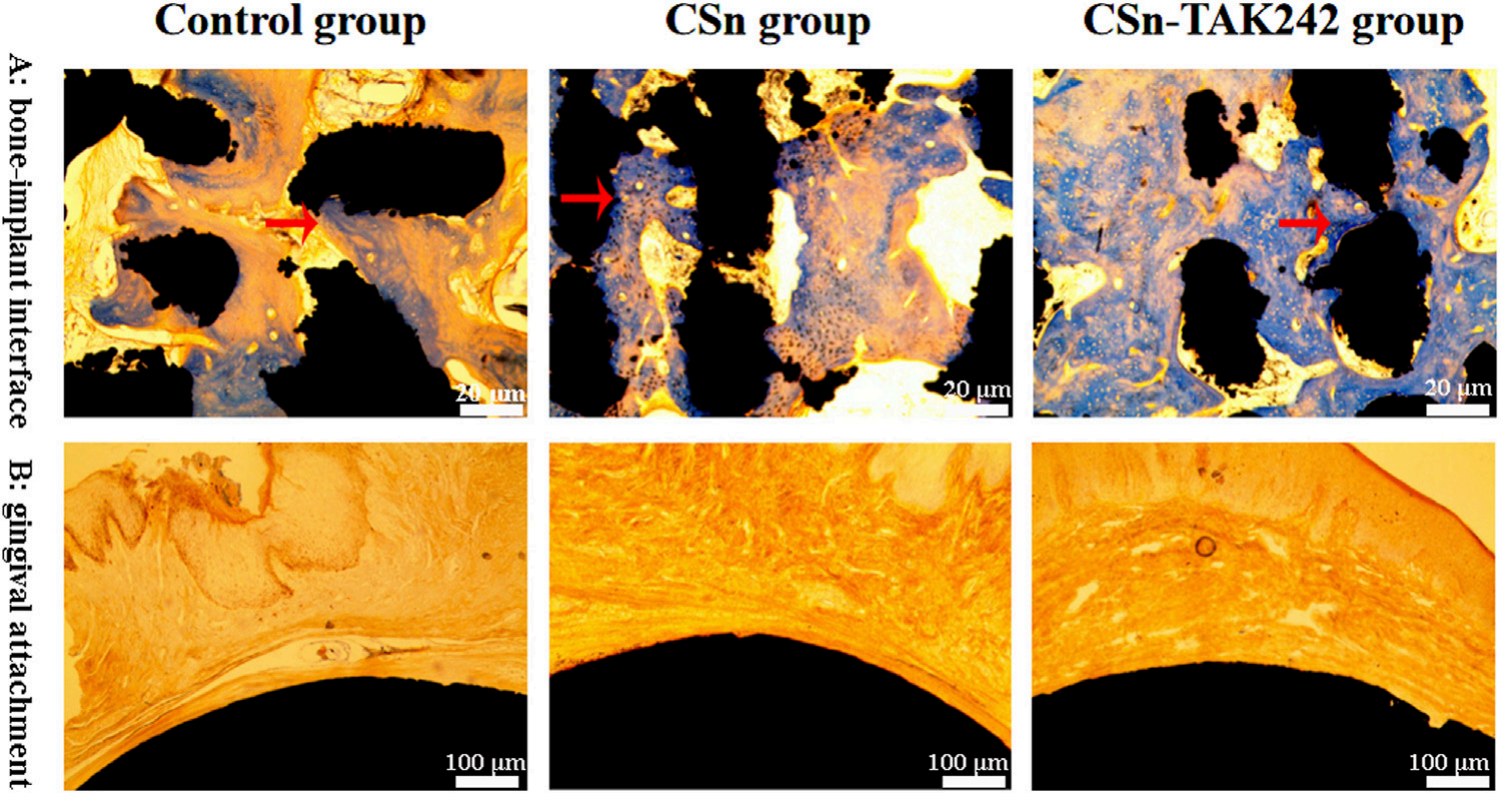
Histological slice observations. **(A)** Observation of osseointegration of implants. **(B)** The adhesion between the smooth arc-shaped structure on the upper part of the implant and the gingival tissue.

The histological sections observed under a microscope 3 months after the implantation of group 3 porous titanium RAIs are shown in Figure 8. Bone tissue is present in the pores inside the implants of the CSn-TAK242 group, CSn group, and Control group, forming a good bone integration tightly connected to the implants. The bone formation in the pores of the CSn-TAK242 group implants is superior to that of the CSn and Control groups. The smooth, rounded structures at the top ends of the implants in all three groups are closely adhered to by gingival tissue.

## 4 Discussion

Currently, the crowns of implant-supported dentures are crafted to replicate the appearance and function of natural teeth, meeting both aesthetic and practical requirements. However, the underlying support structures (typically cylindrical or conical implants) differ markedly from the natural root forms of molars, failing to emulate the biomechanical properties of natural tooth roots. With recent advancements in digital medicine and 3D printing technologies, researchers have started to explore the creation of RAIs, custom-designed to mirror natural tooth roots (Guo et al., 2020). This biomimetic approach aims to harmonize form and function in implant-supported denture restoration, offering a more integrated solution in dental prosthetics.

Compared to the standardized production of traditional cylindrical or conical implants, the advantages of RAIs are primarily: 1) The shape is consistent with the alveolar socket, allowing for extensive contact with the bone walls of the socket to generate friction and thus achieve good initial stability; 2) Often, there is no need for complex preparation of the alveolar socket, avoiding the heat damage to alveolar bone caused by mechanical cutting in traditional implant surgeries; 3) Immediate implantation of RAIs post-extraction can preserve the alveolar bone to the greatest extent, slowing the resorption of the bone walls of the socket, which is crucial for maintaining the morphology of soft and hard tissues in the implant area and reshaping aesthetic outcomes; 4) Reducing the difficulty of immediate implant surgeries and simplifying the surgical procedures, thus shortening the treatment period and alleviating patient discomfort. Customized RAIs effectively address the surgical challenges associated with immediate placement of traditional implants (Xu et al., 2022; Yang et al., 2023). Therefore, this study fabricated porous titanium RAIs consistent with natural tooth roots, coated with CSn and CSn-TAK242 coatings, and verified their osteogenic effects *in vivo* through animal experiments, providing experimental evidence for clinical applications.

Initial stability and the rate of osseointegration are crucial factors determining the success of dental implants. Good initial stability forms the foundation for implant survival, while an excellent rate of osseointegration ensures long-term stability within the alveolar bone. Research has shown that resonance frequency analysis can measure the stability of implants, providing an ISQ ranging from 0 to 100 (Huang et al., 2020). Higher ISQ values indicate better stability, with implants exhibiting ISQ values above 60 having a very low failure rate. In this study, three groups of porous titanium RAIs, consistent with the morphology of the alveolar socket, were immediately implanted, achieving high average ISQ values due to extensive frictional contact with the bone walls of the socket, thus facilitating ideal osseointegration.

Furthermore, studies suggest that the BIC rate should be no less than 50% to ensure the implant can withstand functional loads (Jiang et al., 2015). Micro-CT scans from our study revealed that all three groups of porous titanium RAIs achieved satisfactory osseointegration 3 months post-implantation: the Control group had a BIC of (68.11 ± 3.63)%, the CSn group had (71.07 ± 2.83)%, and the CSn-TAK242 group (78.69 ± 4.52)%. The BIC value of the CSn-TAK242 group was significantly higher than that of the control group, which meant that the coating accelerated the formation of local bone tissue and improved the osseointegration of RAIs. In comparison, similar studies found a BIC of (41.2 ± 20.6)% for pure titanium RAIs after 6 months. Our porous titanium RAIs showed superior osseointegration compared to similar titanium implants. Many scholars have reported on the BIC of standardized production implants; for instance, Freilich and others implanted Straumann implants into rabbit mandibles with DBM osteogenic scaffolds, resulting in a BIC of 58.1% at 8 weeks, and Kim and others reported a BIC ranging from 60.9% to 65.5% 10 weeks after implanting Osstem implants in dogs post-extraction (Kim et al., 2008; Freilich et al., 2009). Compared to these studies, our porous titanium RAIs achieved better osseointegration within a shorter healing period and outperformed standardized production implants. This improvement is due to the suitable porosity of our implants, which enhances the osseointegration rate by increasing the contact area between the implant and bone tissue, thereby facilitating the localization, adhesion, proliferation, and differentiation of bone cells. Bone cells grow into the porous structure, forming micromechanical interlocks with the implant, which increases the strength of osseointegration. Additionally, the interconnected network structure of the pores provides an advantageous environment for the exchange of bodily fluids, promoting bone tissue reconstruction and regeneration, and accelerating the process of osseointegration (Yu et al., 2020). Histological sections observed under the microscope showed that bone integration occurred not only on the outer surface of the porous titanium RAIs but also within the internal pores, confirming that these implants can achieve ideal osseointegration with the alveolar bone in a shorter healing period, thus ensuring their long-term stability.

To enhance the osseointegration between porous titanium RAIs and alveolar bone, this study applied composite CSn coatings and CSn-TAK242 coatings to the implant surfaces. Chitosan, a natural, non-toxic alkaline polysaccharide with excellent biocompatibility, degradability, and antibacterial properties, is widely used in the biomedical field. Chitosan-based drug delivery systems are favored for their targeting, sustained release, improved drug stability, and extended duration of action, often carrying bone metabolic drugs, growth factors, and exogenous regulatory genes to promote bone cell growth and tissue healing (Li et al., 2019; Liu et al., 2024). TAK242, an effective inhibitor of TLR4, reduces the expression of TLR4 and decreases the destructive impact of inflammatory factors on periodontal tissues, while also minimizing their negative effects on osteogenesis, thereby indirectly promoting osseointegration. The CSn-TAK242 coating demonstrated superior osteogenic effects compared to both the CSn coating and uncoated porous RAIs. The TLR4/MyD88 signaling pathway plays a significant role in bone metabolism and immune response regulation in osteolytic diseases. Although studies confirm TLR4’s involvement in the healing of soft and hard tissue injuries, its role in implant osseointegration remains unclear. This study, by inhibiting the TLR4 signaling pathway, aimed to mitigate the inflammatory response and promote implant osseointegration (Zhong et al., 2019). The experimental results validated our hypothesis that inhibiting TLR4 could reduce inflammatory reactions during implantation, decrease alveolar bone resorption, and enhance implant osseointegration. Additionally, applying CSn and CSn-TAK242 coatings on porous titanium RAIs validated their role in enhancing osseointegration in animal experiments, aligning with the preliminary *in vitro* findings. Therefore, this study concludes that chitosan, as an effective bone-inducing material, can be applied as a surface coating on porous titanium RAIs to enhance osseointegration. Moreover, as an efficient drug carrier, chitosan can deliver TAK242 and release it slowly in the osseointegration area, offering superior enhancement of bone integration for porous titanium RAIs. While promising, the clinical translation of this strategy must consider potential limitations, including the immunogenic risk associated with animal-derived chitosan.

TLR4 is a sensitive transmembrane receptor protein that recognizes bacterial products and cellular debris, transducing downstream signals to initiate inflammatory responses. MyD88 and TRIF are two major adaptor proteins mediating TLR4 signal transduction and downstream pathways. The MyD88-dependent pathway serves as the primary signaling route for most Toll-like receptors; however, TLR4 can also indirectly recruit TRIF via the TRAM adaptor molecule. The signaling cascade dependent on TRIF is referred to as the MyD88-independent pathway. Studies have shown that MyD88-knockout mice exhibit accelerated bone healing accompanied by attenuated inflammatory responses compared to wild-type mice. In this model, enhanced bone regeneration may be mediated through the MyD88 pathway. Research also indicates that TRIF mediates multiple signaling cascades, not only triggering the release of various inflammatory factors but also participating in processes such as cell proliferation and apoptosis. Tsutsui et al. (2010) demonstrated that LPS activates the TRIF pathway via TLR4 and upregulates TNF-α expression. Additionally, LPS can stimulate immune cells or organs through the MyD88 pathway to promote TNF-α release, modulate the expression of macrophage colony-stimulating factor and osteoclast differentiation factor, and thereby regulate osteoclast differentiation. The present experimental results revealed that both the CSn and CSn-TAK242 groups exhibited reduced expression levels of MyD88 mRNA and TRIF mRNA. Furthermore, these groups demonstrated significantly improved bone union and milder inflammatory responses compared to the control group. Thus, this study confirms that inhibition of TLR4 function exerts a positive influence on downstream signaling pathways and the process of bone healing.

It is noteworthy that the TLR4 mRNA expression level in the CSn-TAK242 group was significantly higher than that in the CSn group. This phenomenon may be attributed to the specific blockade of the TLR4 intracellular signaling pathway by TAK242, which prevents its interaction with adaptor molecules. However, TLR4 itself is not eliminated; instead, its expression may be upregulated due to negative feedback regulation. Furthermore, the TLR2 mRNA level in the CSn-TAK242 group was higher than those in both the CSn group and the control group, suggesting potential crosstalk between Toll-like receptors. This observation is supported by the findings of Wang et al. (2012), who reported that TLR2 knockout mice exhibited reduced TLR4 mRNA levels compared to wild-type mice, demonstrating that the effects of TLR2 deficiency may be partly mediated through downregulation of TLR4 expression. The results of this experiment are consistent with it.

A total of 48 porous titanium RAIs were placed in this experiment, all of which demonstrated good primary stability and were subjected to submerged healing. However, only seven implants were ultimately retained, indicating a relatively high failure rate. The authors suggest that the loss of porous titanium RAIs may be attributed to multiple factors, potentially related to experimental design, surgical procedures, and postoperative animal care. Furthermore, the missing RAIs will be analyzed and discussed in detail in the follow-up study.

## 5 Conclusion

In summary, the use of 3D-printed porous titanium RAIs has effectively replaced compromised dental structures, achieving a seamless unity of form and function. Meanwhile, CSn-TAK242 coatings on porous titanium RAIs also exhibiting superior osseointegration. However, issues such as the connection methods between porous titanium RAIs and the upper dental crowns, as well as the placement techniques for the implants, will require further exploration and research.

## Data Availability

The raw data supporting the conclusions of this article will be made available by the authors, without undue reservation.
